# Brain Functional Connectivity Plasticity Within and Beyond the Sensorimotor Network in Lower-Limb Amputees

**DOI:** 10.3389/fnhum.2018.00403

**Published:** 2018-10-09

**Authors:** Jingna Zhang, Ye Zhang, Li Wang, Linqiong Sang, Lei Li, Pengyue Li, Xuntao Yin, Mingguo Qiu

**Affiliations:** ^1^Department of Medical Imaging, College of Biomedical Engineering, Army Medical University, Chongqing, China; ^2^Department of Rehabilitation, Southwest Hospital, Army Medical University, Chongqing, China; ^3^Department of Radiology, Southwest Hospital, Army Medical University, Chongqing, China

**Keywords:** lower-limb amputation, functional reorganization, resting-state functional connectivity, the sensorimotor network, S1M1, subcortical nuclei

## Abstract

Cerebral neuroplasticity after amputation has been elucidated by functional neuroimaging. However, little is known concerning how brain network-level functional reorganization of the sensorimotor system evolves following lower-limb amputation. We studied 32 unilateral lower-limb amputees (LLAs) and 32 matched healthy controls (HCs) using resting-state functional magnetic resonance imaging (rs-fMRI). A regions of interest (ROI)-wise connectivity analysis was performed with ROIs in eight brain regions in the sensorimotor network to investigate intra-network changes, and seed-based whole-brain functional connectivity (FC) with a seed in the contralateral primary sensorimotor cortex (S1M1) was used to study the FC reorganization between the sensorimotor region (S1M1) and other parts of the brain in the LLAs. The ROI-wise connectivity analysis showed that the LLAs had decreased FC, mainly between the subcortical nuclei and the contralateral S1M1 (*p* < 0.05, FDR corrected). Seed-based whole-brain FC analysis revealed that brain regions with decreased FC with the contralateral S1M1 extended beyond the sensorimotor network to the prefrontal and visual cortices (*p* < 0.05, FDR corrected). Moreover, correlation analysis showed that decreased FC between the subcortical and the cortical regions in the sensorimotor network progressively increased in relation to the time since amputation. These findings indicated a cascade of cortical reorganization at a more extensive network level following lower-limb amputation, and also showed promise for the development of a possible neurobiological marker of changes in FC related to motor function recovery in LLAs.

## Introduction

With increases in traffic accidents, malignant diseases and natural disasters, the number of lower-limb amputees (LLAs) is increasing (Barbosa et al., 2016). LLAs have difficulties in motor control and coordination, especially in the early stages following amputation because of the major structural asymmetry arising from the amputation (Molina Rueda et al., 2013). With a period of rehabilitation and the application of a prosthesis, the functional status of amputees is gradually restored (Soares et al., 2009; Rusaw and Ramstrand, 2011), which could induce functional reorganization in the sensory and motor areas of the brain.

One of the most prominent consequences of limb amputation concerns the functional reorganization in the primary somatosensory and motor cortices. Several studies in non-human primates have demonstrated that the loss of an upper limb results in functional reorganization of the primary sensorimotor cortex (S1M1) (Kaas et al., 1983; Merzenich et al., 1983; Pons et al., 1991; Jain et al., 2008). In humans, such reorganization after limb amputation has also been observed, and this has been interpreted as a form of maladaptive plasticity in these cortices that is triggered by the loss of sensory inputs (MacIver et al., 2008) or voluntary control (Raffin et al., 2016). However, most of the studies dealing with cortical reorganization included only patients that have undergone upper-limb amputation. The patterns of brain reorganization might vary significantly in LLAs due to differentiation in the functions and representations between the upper and lower limbs. A remapping of the cortical topography was observed in LLAs, with an expansion of activation maps of the stump in the S1M1 of the deafferent hemisphere, spreading to neighboring regions that represent the trunk and upper limbs (Simões et al., 2012), which indicates that lower-limb amputation also induces neuroplastic changes in the S1M1.

Although emphasis has been given to the sensorimotor cortex in the functional reorganization following limb amputation, the contribution of subcortical structures cannot be overlooked (Curt et al., 2011). The cerebellum and the basal ganglia are groups of subcortical nuclei with long-established roles in motor control (Hoshi et al., 2005; Bostan and Strick, 2010; Bostan et al., 2013). Both of these structures are important subcortical structures in the motor circuit, and perform distinct functional operations such as the initiation and execution of voluntary movement (Groenewegen, 2003). However, only a few studies have examined functional and structural alterations of these subcortical nuclei following lower-limb amputation. A fMRI study found increased activation in the contralateral basal ganglia during motor imagery of the amputated toes in LLAs (Romero-Romo et al., 2010). A voxel-based morphometric (VBM) study found decreased gray matter volume in the bilateral cerebellum in LLAs (Di Vita et al., 2018). Structural and functional abnormalities of the thalamus have also been found following peripheral deafferentation. Garraghty and Kaas (1991) found functional reorganization in the thalamus of adult monkeys after peripheral nerve injury. Draganski et al., (2006) showed a significant decrease in the gray matter volume of the thalamus following limb amputation. Results from a study by Jones and Pons (1998) demonstrated that long-standing limb amputation can cause structural reorganization of the thalamus. These findings implied that these movement-related subcortical structures are possibly involved in functional reorganization in LLAs. However, how these subcortical structures work during the process of functional reorganization following lower-limb amputation has not yet been elucidated.

In recent years, functional connectivity (FC) analyses have provided invaluable approaches for studying the human brain in healthy (Smith et al., 2009, 2012; Yeo et al., 2011) and diseased groups (Napadow et al., 2010; Collignon et al., 2013; Rocca et al., 2014) on the brain-network level. Among the varied functional neuroimaging techniques, resting-state fMRI (rs-fMRI) is a promising tool for mapping FC. This method does not require the participants to accomplish complex sensorimotor task during functional neuroimaging acquisition. Resting-state FC analysis has been used in studying the network-level reorganization of FC following arm amputation and has found reduced FC between the cortex associated with the missing hand and the sensorimotor network in amputees (Makin et al., 2015). Regarding lower-limb amputation, one diffusion tensor imaging (DTI) study observed that the mean fractional anisotropy (FA) value of the fibers in region II of the corpus callosum, which connects the premotor area and supplementary motor area (SMA), was significantly reduced, demonstrating that structural connectivity between the bilateral sensorimotor cortex was reorganized in LLAs (Li et al., 2017). To our knowledge, there have been no prior studies of changes in FC in LLAs. Thus, resting-state FC analysis may offer a new way to explore functional network reorganization in the brain after lower-limb amputation.

Besides the regions within the sensorimotor network, abnormal brain reorganization has been found in other regions (Preißler et al., 2013; Jiang et al., 2015; Makin et al., 2015). Specifically, Preißler et al. (2013) found that upper-limb amputees were associated with anatomical alterations in parts of the brain region that belong to dorsal visual stream. Such plasticity was hypothesized to reflect a brain adaptation process to new movement and coordination patterns in operating hand prosthesis. Meanwhile, Jiang et al. (2015) found a significantly lower thickness in the motor-related visual cortex after lower-limb amputation which was presumably related to the degeneration of biological motion perception or tactile motion processing. Additionally, Makin et al. (2015) found that in upper-limb amputees, the missing hand cortex gradually became functionally coupling with the default mode network (DMN) and decoupling with the sensorimotor network over the time since amputation. Such network-level cortical reorganization was supposed to be related to complex perceptual experiences of phantom sensations. Thus we conclude that limb amputation may cause more extensive brain reorganization beyond the changes that occur in the sensorimotor network.

According to the research mentioned above, amputation may result in extensive structural and functional reorganization in the brain. However, very little is known about large-scale network-level reorganization of FC following lower-limb amputation, which requires further investigation. Here, we take these ideas further by characterizing intra-network (within the sensorimotor network) and inter-network (between S1M1 and other parts of the brain) changes following lower-limb amputation using resting-state FC analysis. We hypothesized that the LLAs would show abnormal FC within and beyond the sensorimotor network compared with the healthy controls (HCs); meanwhile, when considering the long duration of recovery for some amputees, we also hypothesized that FC in the LLAs may present progressive changes in relation to the time since amputation. To test these hypotheses, we compared the regions of interest (ROI)-wise connectivity within the sensorimotor network and the seed-based whole-brain FC of the sensorimotor network between the LLAs and HCs. Further, we investigated the relationship between FC and time since amputation in LLAs.

## Materials and Methods

### Participants

Thirty-two individuals (23 male and 9 female) with acquired unilateral lower-limb amputation (mean age ± SD: 46.03 ± 12.04, 13 patients with amputations on the right side, see **Table 1**) were recruited through the Prosthetic and Orthotic Clinics at the Department of Rehabilitation, Southwestern Hospital (Chongqing, China) between March 2015 and April 2016. Nineteen amputations occurred at the transfemoral level and 13 at the transtibial level. All the patients had been fitted with prostheses. Twenty-five patients underwent amputation following a traumatic injury, and the other amputations were due to tumors (three melanoma, two osteosarcoma, and two osteomyelitis). Phantom limb pain (PLP) was assessed using a 10 cm Visual Analog Scale (VAS) (0: “no pain at all” represented by the left end point and 10: “the strongest pain I can imagine” represented by the right end point of the scale (Scott and Huskisson, 1976). Exclusion criteria were the following: (1) amputation at another part of the body, (2) history of brain injury due to trauma, (3) presence of neurological or psychiatric disorders, (4) presence of stump pain, and (5) duration between amputation and MRI scanning of less than 3 months. Thirty-two age- and sex-matched HCs without neurological or psychiatric diseases and with normal brain MRI (no brain atrophy, tumor, ischemia, hemorrhage, or congenital abnormalities) were recruited from the local community. All subjects were right-handed, as assessed using the Edinburgh Handedness Inventory (Oldfield, 1971). Each participant was informed of the purpose and methods of the study and provided a written consent form prior to MRI. All experimental procedures were approved by the Ethics Committee of the Army Medical University.

**Table 1:** Demographic and clinical characteristics of the participants.

Characteristic	LLAs (*n* = 32)	HCs (*n* = 32)	t/χ^2^	*p-*Value
Age (years)	46.0 ± 12.0 (20–62)	46.0 ± 11.9 (20–62)	0.001	0.972^b^
Education (years)	8.9 ± 2.3	9.1 ± 3.1	-0.454	0.687^b^
Sex (female)	32 (9)	32 (13)	1.108	0.292^a^
Time since amputation (months)	127.7 ± 102.2 (3–396)	–		–
Age at amputation (years)	35.3 ± 12.4 (18–61)	–		–
Amputation at left/right	19/13	–		–
Amputation at femur/tibia	19/13	–		–


*LLAs, lower-limb amputees; HCs, healthy controls. Data are expressed as mean ± SD (the range from minimum–maximum). ^a^The p-value was obtained using Chi-squared test. ^b^The p-value was obtained using two-tailed two independent sample t-test.*

### Image Acquisition

A Siemens 3.0-T Trio MRI scanner (Magnetom Trio, Siemens, Erlangen, Germany) was used to acquire the functional and structural data. Rs-fMRI data were collected via a multi-slice, gradient-echo, echo-planar sequence for a total of 240 volumes (480 s) using the following parameters: repetition time (TR) = 2000 ms; echo time (TE) = 30 ms; flip angle = 90°; matrix = 64 × 64; field of view = 200 mm; slice thickness = 3 mm, 1-mm gap, 36 slices with a voxel size = 3 mm × 3 mm × 3 mm. During the resting-state scan, subjects were asked to relax with their eyes closed and not to think of anything in particular. High-resolution three-dimensional T1-weighted structural images were also collected in the sagittal orientation via a magnetization-prepared rapid gradient-echo sequence (MP-RAGE) using the following parameters: TR = 1,900 ms, TE = 2.52 ms, flip angle = 9°, matrix = 256 × 256, thickness = 1.0 mm, 176 slices with a voxel size = 1 mm × 1 mm × 1 mm.

### Data Preprocessing

Before data preprocessing, in order to obtain a ‘homogeneous’ sample of patients with a left side amputation, we flipped the imaging data from right to left along the midsagittal line for the 13 patients with an amputation on the right side. For all patients, the left side corresponded to the cerebral hemisphere ipsilateral to the side of the amputation and the right side corresponded to the cerebral hemisphere contralateral to the side of the amputation.

Preprocessing was performed using the Data Processing Assistant for Resting-State fMRI (DPARSF) toolbox^1^. The first 10 images of each subject were discarded to allow the signal to reach equilibrium and the participants to adapt to the scanning noise. Then, the remaining 230 images were corrected for temporal delay in slice acquisition and co-registered to the first image for correction of rigid- body head motion. For all subjects, the translation or rotation parameters did not exceed ± 2.5 mm or ± 2.5°, respectively. Since rs-fMRI data have been demonstrated to be sensitive to head motion (Yan et al., 2013a), the head motion effects were regressed using the Friston 24-parameter model (Satterthwaite et al., 2013; Yan et al., 2013a). To further reduce the effects of confounding factors, including the signals from the white matter and cerebrospinal fluid, the mean time series of all voxels across the whole brain were removed from the data via linear regression (Yan et al., 2013b). The resulting maps were spatially normalized into a standard stereotaxic space at a resolution 3 mm × 3 mm × 3 mm using an EPI template. After normalization, the images were smoothed using a full-width at half-maximum Gaussian kernel of 6 mm to increase the signal-to-noise ratio. Finally, a temporal filter (0.01–0.08 Hz) was applied to reduce low-frequency drift and high-frequency physiological noise.

### Seed Selection and FC Analysis

**FIGURE 1:**
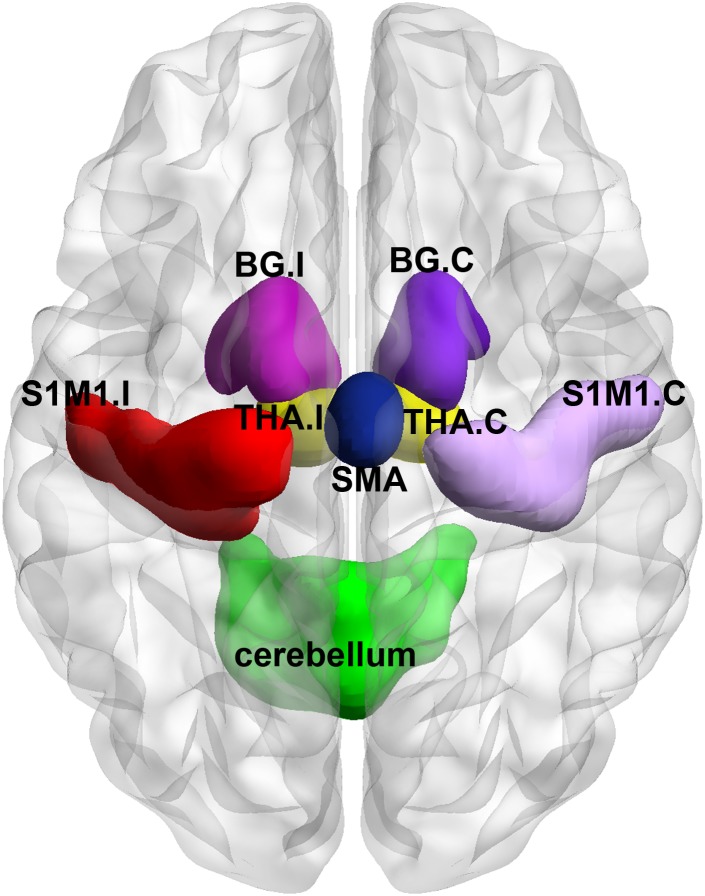
Visual depiction of the regions of interest (ROIs) within the subcortical and cortical sensorimotor network that were used in the analyses described in the text. BG, basal ganglia; THA, thalamus; S1M1, the primary sensorimotor cortex; SMA, the supplementary motor area; I indicates ipsilateral to the side of the amputation in the lower-limb amputees (LLAs); C indicates contralateral to the side of the amputation in the LLAs. I corresponds to the left brain in the healthy controls (HCs), and the C corresponds to the right brain in the HCs.

For ROI-wise connectivity, we selected eight subcortical and cortical sensory- and motor-related brain regions based on a freely available atlas of regions defined by correlated activation patterns^2^ (Shirer et al., 2012), including the bilateral S1M1 with peak Montreal Neurological Institute (MNI) coordinates of (-54, -12, 34) and (58, -10, 34), a bilateral region of the SMA with a peak MNI coordinates of (6, -10, 52), a bilateral region of the cerebellum with a peak MNI coordinates of (-24, -40, -36), the bilateral basal ganglia with peak MNI coordinates of (-22, 2, -6) and (24, 4, -4), and the bilateral thalamus with peak MNI coordinates of (-8, -16, -2) and (10, -16, -2) as the ROIs. In this atlas, the basal ganglia and thalamus on each side of the brain are divided into an entire ROI. A previous study (Draganski et al., 2006) has demonstrated thalamic structural variations due to a chronic lack of afferent input in LLAs, reflecting the role of the thalamus in structural reorganization and functional restoration after injury to the central and peripheral nervous system. Therefore, we considered the thalamus as an independent ROI. In this study, the WFU_Pickatlas toolbox^3^ was used to define the thalamus as an independent ROI, and the remaining part in the original region from the atlas was defined as the ROI of the basal ganglia (Guan et al., 2017). All the ROIs of the subcortical and cortical sensorimotor network were visualized with BrainNet Viewer^4^ (Xia et al., 2013) (**Figure 1**). For simplicity, in the following sections, the brain region contralateral to the side of the amputation was called the contralateral region, and the brain region ipsilateral to the side of the amputation was called the ipsilateral region in LLAs. For example, the S1M1 contralateral to the side of the amputation was called the contralateral S1M1. ROI-wise connectivity was measured by extracting the residual time course data from each ROI listed above and calculating the Pearson’s correlation coefficients between each ROI pair. The resulting correlation coefficients were then Fisher-transformed into ’Z’ scores to increase normality.

To further elaborate inter-FC of the sensorimotor network, we calculated seed-based whole-brain voxel-wise FC (Jung et al., 2013; Fan et al., 2017). The contralateral S1M1 in the LLAs was selected as the seed because it is the most prominent brain region for functional reorganization following amputation (MacIver et al., 2008; Simões et al., 2012; Raffin et al., 2016). The whole-brain resting-state FC maps were obtained for the right S1M1 in HCs and the contralateral S1M1 in patients using the DPARSF toolbox.

### Statistical Analysis

#### Analysis of Demographic and Clinical Characteristics

Two-tailed two sample *t*-tests and chi-square tests (only for sex) were performed to compare the demographic and clinical data from the two groups (SPSS 20.0; SPSS, Inc., Chicago, IL, United States). The significance level was set at *p* < 0.05.

#### Analyses of FC

Analyses of the ROI-wise connectivity were performed using the GRETNA toolbox^5^. Two-tailed two-sample *t*-tests were conducted to identify the specific group differences (*p* < 0.05, FDR corrected). Age, sex, and years of education were controlled as covariates to ensure that our results only revealed FC differences uniquely associated with amputation.

For the FC maps (seed-based whole-brain voxel-wise connectivity), two-tailed two-sample *t*-tests were performed to examine group-related differences between the LLAs and the HCs. Significance thresholds for *t*-test were set at *p* < 0.05 (FDR corrected). The covariates age, sex, and educational level were controlled. These statistical analyses were carried out with the SPM toolbox (spm8^6^).

#### Relationships Between FC and Clinical Characteristics

We correlated clinical characteristics with brain FC, corresponding to the significant group-related difference between the LLAs and the HCs. A two-tailed partial correlation analysis was used after controlling for age, sex, educational level as confounding variables (*p* < 0.05).

## Results

### Demographic and Clinical Characteristics

**Table 1** shows the demographic and clinical characterization of patients with an amputation and the HCs. No significant differences in age, sex, or education were observed between the two groups (*p* > 0.05). PLP was present in 12 LLAs [ranging in pain intensity from 1.1 to 6.8 (3.46 ± 2.19)].

### ROI-Wise Connectivity Within the Sensorimotor Network

**FIGURE 2:**
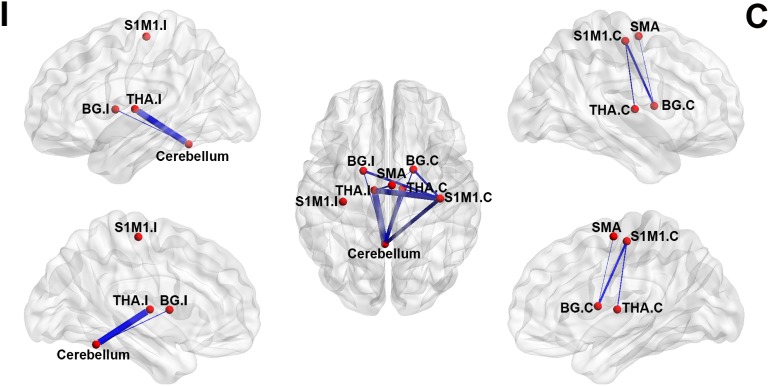
Significant between-group differences in the sensorimotor network in the intensity of the brain FC. The brain networks were visualized with the BrainNet Viewer. The thickness of the line represents the intensity of the between-group difference. BG, basal ganglia; THA, thalamus; S1M1, the primary sensorimotor cortex; SMA, the supplementary motor area; I indicate ipsilateral to the side of the amputation in the LLAs; C indicates contralateral to the side of the amputation in LLAs.

For ROI-wise connectivity, compared with the HCs, the LLAs exhibited significantly decreased FC between the contralateral S1M1 and the bilateral basal ganglia, bilateral thalamus, and cerebellum, between the cerebellum and the bilateral basal ganglia and bilateral thalamus, and between the SMA and the ipsilateral thalamus and contralateral basal ganglia (*p* < 0.05, FDR corrected) (**Figure 2** and **Table 2**).

**Table 2:** Regions of interest (ROI)-wise FC differences within the sensorimotor network between the LLAs and the HCs.

ROI wise FC	LLAs (*n* = 32)	HCs (*n* = 32)	*Z*-value	*p*-value
S1M1.C-BG.I	0.377 ± 0.398	0.683 ± 0.309	-2.932	0.0047
S1M1.C-BG.C	0.380 ± 0.339	0.630 ± 0.354	-2.883	0.0054
S1M1.C-Thalamus.I	0.353 ± 0.315	0.639 ± 0.340	-3.489	0.0009
S1M1.C-Thalamus.C	0.420 ± 0.284	0.636 ± 0.352	-2.688	0.0092
S1M1.C-Cerebelum	0.552 ± 0.367	0.843 ± 0.371	-3.170	0.0024
Cerebelum-BG.I	0.625 ± 0.319	0.826 ± 0.288	-2.664	0.0098
Cerebelum-BG.C	0.561 ± 0.318	0.783 ± 0.341	-2.695	0.0090
Cerebelum-Thalamus.I	0.572 ± 0.280	0.839 ± 0.308	-3.623	0.0006
Cerebelum-Thalamus.C	0.594 ± 0.307	0.845 ± 0.326	-3.170	0.0024
SMA-BG.C	0.386 ± 0.235	0.554 ± 0.283	-2.587	0.0112
SMA-Thalamus.I	0.380 ± 0.227	0.552 ± 0.265	-2.795	0.0069


*FC, functional connectivity; LLAs, lower-limb amputees; HCs, healthy controls; S1M1, the primary sensorimotor cortex; BG, basal ganglia; SMA, the supplementary motor area. I indicates ipsilateral to the side of the amputation. C indicates contralateral to the side of the amputation. Data are expressed as mean ± SD. Z: variables of ROI wise FCs were tested by two-tailed two sample *t*-tests (results were indicated by Z). *p*, statistical significance (*p* < 0.05, FDR corrected).*

### Seed-Based Whole-Brain FC

**FIGURE 3:**
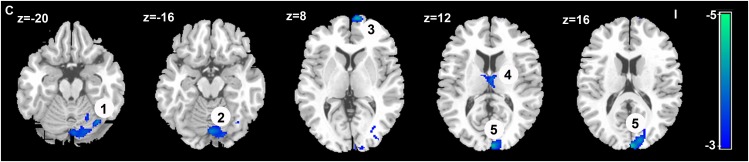
Decreased FC in patients with lower-limb amputation with the seed region in the contralateral S1M1, compared with connectivity in the HCs (*p* < 0.05, FDR corrected, voxels > 50). 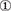: Cerebellum VI; 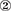: Cerebellum Crus1; 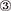: Superior frontal gyrus (medial); 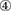: Thalamus; 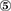: Superior occipital Gyrus, Cuneus, and Middle occipital gyrus. All these brain regions located on the cerebral hemisphere ipsilateral to the side of the amputation. The blue-to-green patches show, on axial slices, the *t* statistic of the comparisons between FC in the LLAs and the HCs. Slice location (z) is displayed in Montreal Neurological Institute coordinates. I indicates ipsilateral to the side of the amputation; C indicates contralateral to the side of the amputation.

The seed-based analysis provides an opportunity to validate and extend the main results obtained in the ROI-wise connectivity analysis, as it allows us to identify which specific loci might underlie the changes in FC between the contralateral S1M1 and the brain regions within and beyond the sensorimotor network in LLAs. When comparing connectivity maps between amputees and the HCs, significantly decreased FC was found between the contralateral S1M1 seed and the ipsilateral cerebellum and the ipsilateral thalamus in the LLAs, which is consistent with the results of the ROI-wise connectivity analysis described above. In addition, the seed-based map in **Figure 3** also shows decreased FC between the contralateral S1M1 and the ipsilateral superior frontal gyrus (medial) and the ipsilateral occipital lobe in LLAs (*p* < 0.05, FDR corrected) (**Figure 3** and **Table 3**).

**Table 3:** Brain regions showing decreased FC with the seed in the contralateral S1M1 in LLAs.

Brain regions	Cluster size (voxels)	Peak coordinates (x/y/z; MNI)			Peak *Z*-value
Cerebellum VI	68	-36	-66	-20	-4.0206
Cerebellum Crus1	238	-2	-78	-16	-3.8483
Superior frontal gyrus (medial)	83	-12	68	4	-4.215
Thalamus	55	-4	-10	12	-3.7372
Superior occipital gyrus, Cuneus, Middle occipital gyrus	230	-6	-98	14	-4.3179


*FC, functional connectivity; LLAs, lower-limb amputees; HCs, healthy controls; S1M1, the primary sensorimotor cortex. All the brain regions showing decreased FC with the seed in the contralateral S1M1 in LLAs located on the cerebral hemisphere ipsilateral to the side of the amputation. x, y, z, coordinates of peak locations in the Montreal Neurological Institute space. *p* < 0.05, FDR corrected.*

To test for possible hemisphere-specific effects, all the calculations were performed on the patients with only right- or left-sided amputations separately. The results did not reveal hemisphere specific differences and therefore all the patients were included in one calculation with a virtual left side amputation in this study. Furthermore, to detect the possible effects of the PLP, we divided all the patients into two groups (LLA with PLP and LLA without PLP). We compared the ROI-wise and the seed-based FC between two groups and failed to find any significant differences between the two groups.

### Correlations Between FC and Clinical Characteristics

**FIGURE 4:**
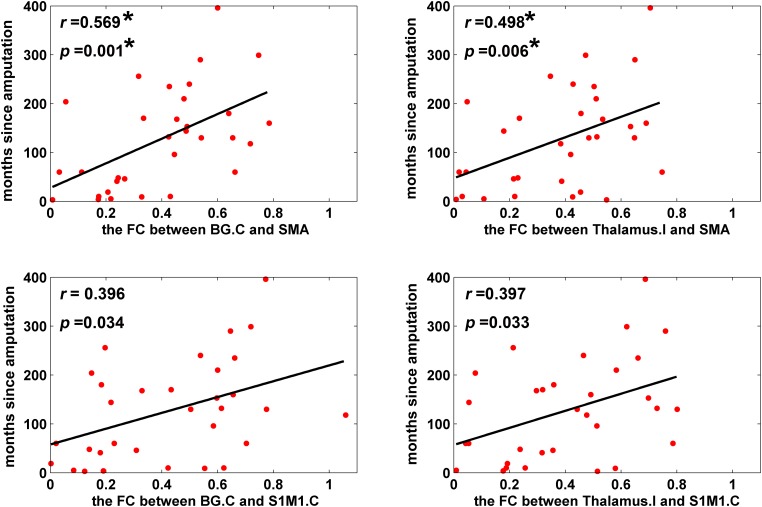
Scatter plots depicting the correlations between FC and time since amputation (*p* < 0.05). Partial correlation coefficients (*r*) were corrected for age, sex, and education. Scatter plots is fitted with regression line (black line). ^∗^The correlations survived a critical FDR threshold. BG, basal ganglia; SMA, the supplementary motor area; S1M1, the primary sensorimotor cortex; I indicates ipsilateral to the side of the amputation; C indicates contralateral to the side of the amputation.

For ROI-wise connectivity, as shown in **Figure 4**, partial correlation analysis revealed that the FC between the SMA and the contralateral basal ganglia and ipsilateral thalamus and between the contralateral S1M1 and the contralateral basal ganglia and ipsilateral thalamus was positively correlated with the time since amputation in LLAs (*p* < 0.05, uncorrected). Only the correlations between the time since amputation and FC between the SMA and the contralateral basal ganglia and ipsilateral thalamus survived after FDR correction for multiple comparisons (*p* < 0.05, FDR corrected). Meanwhile, partial correlation analysis showed that the extracted FC of the cluster (the contralateral S1M1 seed to the ipsilateral thalamus) identified in the group comparison of seed-based FC was significantly correlated with the time since amputation in LLAs (*r* = 0.417, *p* = 0.017).

## Discussion

Accumulating evidence has indicated that amputation induces functional reorganization of the sensorimotor areas in the brain, including the S1M1, cerebellum, thalamus, and basal ganglia, which could be related to abnormal functioning of the subcortical-cortical sensorimotor system resulting from the reduction or absence of afferent information following amputation (Draganski et al., 2006; Romero-Romo et al., 2010; Simões et al., 2012; Raffin et al., 2016; Di Vita et al., 2018). In addition, structural and functional reorganization of the brain has also been found in the visual stream (Preißler et al., 2013; Jiang et al., 2015) and the DMN (Makin et al., 2015), which provides evidence of brain reorganization beyond the sensorimotor system. However, there have been few studies on patterns of reorganization in brain functional networks, especially in patients with lower-limb amputation. Moreover, previous studies have found progressive changes in brain structure and function after amputation (Jiang et al., 2015, 2016; Li et al., 2017), which suggests the need for further exploration of the relationship between imaging indicators and time since amputation.

In the current study, we investigated changes in the organization of the intra- and inter-FC of the sensorimotor network in unilateral LLAs, and the relationship between FC and the time since amputation. We identified abnormal FC within and beyond the sensorimotor network. In addition, we found positive correlations between FC among subcortical and cortical sensorimotor regions and the time since amputation.

### Altered FC Within the Sensorimotor Network

As a drastic limb injury, amputation in humans induces changes in the S1M1 of the deafferent hemisphere (Ramachandran et al., 1992; Lotze et al., 1999; Giraux et al., 2001; Karl et al., 2001; Grüsser et al., 2004; Simões et al., 2012; Raffin et al., 2016). In this study, decreased FC among the contralateral S1M1 and the subcortical motor-related brain regions was found in LLAs by using an ROI-wise connectivity analysis. Our findings provide further evidence of network-level functional reorganization of the S1M1. We also found abnormal FC among the ipsilateral thalamus, the contralateral basal ganglia and the SMA in LLAs. To our knowledge, no prior study has reported the SMA function reorganization in amputees. A previous study found that the motor imagery of both normal and amputated limb can activate the SMA, and there was no significant difference in the intensity and range of activation in upper-extremity amputees (Diers et al., 2010). As an important part of the sensorimotor network, the SMA regulates the balance of the bilateral body and controls motion (Penfield and Welch, 1951; Serrien et al., 2002), playing an especially important role in controlling the movement of the lower limb (Luft et al., 2002; Sahyoun et al., 2004). Previous studies showed that SMA activation was prominent for parts of lower limb, such as the knee (Luft et al., 2002), and brain activation of the SMA was associated with gait control (Sahyoun et al., 2004). Therefore, changes in the FC of the SMA may be a unique pattern of brain reorganization in patients with lower-limb amputation.

In addition to cortical areas, the sensorimotor network consists of subcortical areas such as the cerebellum, basal ganglia and thalamus (Middleton and Strick, 2000; Dum and Strick, 2002). A previous study suggested that the cerebellum plays a role in the generation of appropriate patterns of limb movements, dynamic regulation of balance, and adaptation of posture and locomotion (Morton and Bastian, 2004). In addition, the cerebellum and the basal ganglia, as the major centers of the extra pyramidal motor system, exert their regulatory influence on the motor cortex via the thalamus to ‘shape’ the final automatic and voluntary output movement (Herrero et al., 2002; Groenewegen, 2003; Mizelle et al., 2016). All these findings reflect the essential role of these subcortical areas in the organization of limb movements. The structural and functional reorganization in these subcortical nuclei have been corroborated in neuroimaging studies of patients who have undergone lower-limb amputation. Recent work reported cerebellar gray matter modifications in LLAs (Di Vita et al., 2018), and a lower volume in the thalamus was found in subjects with limb amputation (Draganski et al., 2006). Moreover, Romero-Romo et al. (2010) found a disrupted basal ganglia-thalamus-cortex pathway in LLAs when the amputee group performed imaginary movements of amputated toes. In our study, we chose these subcortical areas as the ROIs to study the sensorimotor network in the LLAs and found decreased FC between the subcortical nuclei and between the subcortical nuclei and the SMA and the contralateral S1M1. The changes of FC in the brains of LLAs revealed the abnormal subcortical-cortical sensorimotor network after amputation, further highlighting the important role of subcortical areas in the reorganization of the sensorimotor network after lower-limb amputation.

### Altered FC Between the S1M1 and the Other Parts of the Brain

The seed-based analysis also revealed aberrant connectivity within the sensorimotor network in LLAs. We found that the FC between the contralateral S1M1 and the ipsilateral cerebellum and the ipsilateral thalamus was reduced. These results confirmed the functional reorganization of the sensorimotor network in the patients with lower-limb amputation. In addition, we found reduced FC between the contralateral S1M1 and the ipsilateral superior frontal gyrus (medial) in the LLAs. The superior frontal gyrus (medial) is a part of the DMN (Greicius et al., 2003; Buckner et al., 2008; Raichle, 2015), which is involved with the representation of internalized sensations. Abnormal FC between the region of the cortex associated with a missing hand and the DMN was found in patients with arm amputation, which was suggested to correlate with complex perceptual experiences associated with phantom sensations (Makin et al., 2015). Meanwhile, we found reduced FC between the contralateral S1M1 and the ipsilateral occipital lobe, including the superior occipital gyrus, cuneus and middle occipital gyrus, which are important for visual processing. Previous fMRI studies found that amputation of the hand leads to shifts in visuospatial perception (Makin et al., 2010), as well-reductions in visuomotor object affordances (Wilf et al., 2013). Moreover, decreased cortical thickness was also reported in visual areas in LLAs (Jiang et al., 2015). Indeed, in the healthy brain, visual information of the limb is integral for motor processing during movement (Makin et al., 2014). The reduction of FC between visual and motor areas proved that in addition to visual cortical abnormalities, there was also an abnormal visual-motor information processing loop in LLAs. These findings demonstrated that functional consequences of amputation may result from reorganization at a network-level scale beyond the sensorimotor network. In this study, all the patients had been fitted with prostheses. The confounding effects of prosthesis use should be considered in the context of these results, as they may be associated with the brain functional reorganization of the brain in LLAs.

### The Associations Between the Time Since Amputation and the FC

Our correlation analyses revealed statistically significant positive associations between the time since amputation and the FC in the contralateral basal ganglia, ipsilateral thalamus and SMA, and the contralateral basal ganglia, ipsilateral thalamus and contralateral S1M1 in the LLAs, which suggested that there were progressive subcortical-cortical FC changes in LLAs. Recent MRI studies have demonstrated that the brain changes over time following amputation (Jiang et al., 2015, 2016; Li et al., 2017). However, the majority of these studies mainly focused on the structural abnormalities of the LLAs. Jiang et al. (2015) found that LLAs displayed significant FA reduction in the right inferior front occipital fasciculus and a reduced thickness of the motor-related visual cortex (Jiang et al., 2016), both of which were negatively correlated with the time since amputation. Li et al. (2017) found that mean FA values of the corpus callosum were significantly smaller in amputees and that values were correlated with the time since amputation. These previous findings suggest a progressively disrupted pattern in the brain gray matter and white matter over the time following amputation. In this study, we found progressive increases in the reductions of connectivity between the brain subcortical-cortical regions in the LLAs, which could be a form of compensation for the increase in serious structural damage over time. Another possible explanation for this restoration of FC is that the functional network reorganization after amputation is more likely to be reversed/modulated by motion exercises or prosthesis use. Future rehabilitation treatments should pay attention to the progressive changes in the FC of the sensorimotor network, and the gradual progression of FC may be an important indicator for evaluating the rehabilitation of motor function in patients following lower-limb amputation.

### Limitations

Despite of the important results showing brain functional network reorganization in the LLAs mentioned above, several limitations in this study should be further considered. First, in the current study, we mainly choose the sensorimotor network to study the functional reorganization process over time since amputation, which limits our ability to speculate on global brain organization patterns after amputation. Second, the sample size of the LLAs is relatively small. Studies with larger sample sizes are needed to clarify the specific functional reorganization mechanisms in patients with different types of amputations and to disentangle the influences of the amputation site, the amputation side and the use of prostheses on cortical reorganization in amputees. Third, previous studies found that PLP may affect brain functional reorganization (Flor et al., 2006) and could have an impact on perceived motor control in the amputees (Kikkert et al., 2017). However, we did not find significant differences between LLA with PLP and LLA without PLP in our study. It may be attributed to a small number of amputees with PLP and a greater number with low intensity PLP in this study. In future studies, a large number of amputees with different PLP levels are needed to further study the effects of PLP on brain functional reorganization in the LLAs. Meanwhile, phantom sensations are not assessed in our study. This information needs to be supplemented to investigate the effects of phantom sensations on brain functional reorganization in the LLAs in a subsequent study.

## Conclusion

Our results revealed that lower-limb amputation leads to subcortical and cortical reorganization in the sensorimotor network. Moreover, between-group differences in the seed-based whole-brain FC analysis showed that brain regions with decreased FC with the contralateral S1M1 extended to the prefrontal [superior frontal gyrus (medial)] and occipital cortices. These findings suggested that functional reorganization after lower-limb amputation occurred at multiple scales, within and beyond the scope of the sensorimotor network. Finally, correlation analysis results showed that decreased FC between the subcortical and the cortical regions (the SMA and the contralateral S1M1) progressively increased over the time since amputation, which reflects the process of brain functional network recovery, probably related to motion exercises or prosthesis use after amputation. All of these results hold promise for developing reliable biomarkers of FC changes related to motor function recovery in LLAs.

## Ethics Statement

We declare that all human studies have been approved by the Army Medical University Ethics Committee and have therefore been performed in accordance with the ethical standards laid down in the 1964 Declaration of Helsinki and its later amendments. We declare that all patients gave informed consent prior to inclusion in this study.

## Author Contributions

JZ designed and performed the experiments, analyzed the data, and wrote the manuscript. LW and LS assisted in fMRI data analysis. YZ assisted in writing and editing the manuscript. LL and PL assisted in sample collection and clinical data analysis. XY and MQ provided overall project supervision, helped to analyze and interpret the data, and assisted in writing the manuscript. All authors have read and approved the final manuscript.

## Conflict of Interest Statement

The authors declare that the research was conducted in the absence of any commercial or financial relationships that could be construed as a potential conflict of interest. The reviewer MS and handling Editor declared their shared affiliation.

## Footnotes

1 http://rfmri.org/DPARSF

2 http://findlab.stanford.edu/functional_ROIs.html

3 https://www.nitrc.org/projects/wfu_pickatlas

4 http://www.nitrc.org/projects/bnv/

5 https://www.nitrc.org/projects/gretna/

6 http://www.fil.ion.ucl.ac.uk/spm/software/spm8/
